# Genetic variation in leptin and leptin receptor genes is a risk factor for idiopathic recurrent spontaneous abortion

**DOI:** 10.3325/cmj.2016.57.566

**Published:** 2016-12

**Authors:** Andrijana Müller, Jasenka Wagner, Alenka Hodžić, Aleš Maver, Ivana Škrlec, Marija Heffer, Lada Zibar, Borut Peterlin

**Affiliations:** 1 Department of Gynecology and Obstetrics, University Hospital Center Osijek, Osijek, Croatia; 2 Department of Medical Biology and Genetics, Faculty of Medicine, Josip Juraj Strossmayer University of Osijek, Osijek, Croatia; 3 Clinical Institute of Medical Genetics, Division of Obstetrics and Gynecology, University Medical Center Ljubljana, Ljubljana, Slovenia; 4 Department for Dialysis, Internal Clinic, University Hospital Center Osijek, Osijek, Croatia; 5 Faculty of Medicine, Josip Juraj Strossmayer University of Osijek, Osijek, Croatia

## Abstract

**Aim:**

To determine whether maternal leptin (*LEP*) and leptin receptor (*LEPR*) gene polymorphisms are associated with idiopathic recurrent spontaneous abortion (IRSA).

**Methods:**

This case-control association study conducted from 2010 to 2012 at the Department of Gynecology and Obstetrics, University Hospital Center Osijek and Clinical Institute of Medical Genetics Ljubljana included 178 women with a history of three or more IRSAs before the 22nd week of gestation and 145 women with at least two live births and no history of pathologic pregnancies during reproductive period. Polymorphisms of maternal *LEP* (rs7799039, rs2122627, rs11761556, rs10244329) and *LEPR* (rs1137101, rs7516341, rs1186403, rs12062820) were assessed by allele specific real-time polymerase chain reaction. Genotype distribution, allele frequencies, and frequency of haplotypes at *LEP* and *LEPR* genetic loci were determined.

**Results:**

We observed more frequent genotype for rs7516341 (nominal *P* = 0.034, odds ratio [OR] 0.61, 95% confidence interval [CI] 0.38-0.97) and rs1137101 (nominal *P* = 0.048, OR 1.66, 95% CI 1.00-2.80) in the *LEPR* gene in patients than in controls, but these results did not remain significant after correction for multiple testing according to Bonferroni (adjusted *P* value threshold was set at 0.05). We did not observe differential distribution of genotype frequencies in the *LEP* gene between cases and controls. In patients with IRSA, GTCC haplotype in the *LEPR* gene locus was significantly less frequent than in controls (*P* = 0.00865, OR 0.45), contrary to ACTC haplotype (*P* = 0.0087, OR 1.98).

**Conclusions:**

We showed that genetic variability in the *LEPR* gene was associated with IRSA, warranting confirmation on a greater number of patients and pathogenesis investigation.

Recurrent spontaneous abortion (RSA) is a spontaneous loss of three or more pregnancies before the 22nd week of gestation. It affects approximately 0.5%-3% of women and can be caused by several factors (1). However, about 50% of the cases remain unexplained and classified as idiopathic (IRSA) (2).

Among the factors that raise the risk of several pregnancy complications, including the risk of recurrent miscarriage, is obesity (3). One of the mechanisms involved in obesity is associated with mutations in leptin signaling pathway; mutations in leptin (*LEP*) and leptin receptor (*LEPR*) gene are associated with obesity in rodents and humans (4-6). Also, leptin affects the balance of cytokines in the feto-placental unit (7), and therefore the pregnancy outcome (8,9). Also, leptin synthesized in the placenta acts through binding to leptin receptors, which are expressed in the trophoblast (10,11).

Previous research has shown that genetic variability in maternal *LEP* and *LEPR* gene might be involved in pathological processes in pregnancy, including preeclampsia and gestational diabetes mellitus (12,13), while the role of the *LEP* and *LEPR* gene in the IRSA has not been investigated. Therefore, we hypothesized that maternal genetic variability of the *LEP* and *LEPR* genes was associated with IRSA. For this purpose we performed a case-control genetic association study of polymorphic sites in these two genes on a population of patients with IRSA in comparison with population of women with two or more live births and no history of pathologic pregnancies.

## Materials and methods

### Participants

The study included 323 women. The IRSA group consisted of 178 women (consecutive patients) with a history of three or more spontaneous abortions of unknown etiology before the 22nd week of gestation. The median of age was 33 years (range, 23 to 46) in the IRSA group and 34 years (range, 20 to 48) in controls. All patients had normal karyotypes and no history of endocrine, metabolic, autoimmune, or other systemic disorders, venous or arterial thrombosis, or uterine anatomic abnormalities. The control group consisted of 145 women with at least two normal pregnancies and no pregnancy loss or any other pregnancy related complications. The miscarriages were not investigated cytogenetically. Participants were of Caucasian origin, recruited at the Department of Obstetrics and Gynecology, Clinical Hospital Center Osijek, and the Clinical Institute of Medical Genetics, Division of Obstetrics and Gynecology, University Medical Center, Ljubljana, Slovenia from 2010 till 2012 (Table 1). The study was approved by Slovenian and Croatian National Ethics' Committees, and each of the patients provided written informed consent.

**Table 1:** Characteristics of patients with idiopathic recurrent spontaneous abortion (IRSA)

	IRSA
Age (years), median (range)	33 (23-46)
Parity, n (%)	
0	118 (66.3)
1	45 (25.3)
2	15 (8.4)
Spontaneous abortions, n (%)	
3	162 (91)
4	8 (4.5)
5	7 (3.9)
10	1 (0.5)
Duration of gestation, n (%)	
first trimester	162 (91.9)
second trimester	12 (8.1)

### Single nucleotide polymorphisms (SNPs) selection and genotyping

We genotyped genetic variants in *LEP* and *LEPR* gene. Eight tagging SNPs were chosen, four SNPs in *LEP* gene – rs7799039, rs2122627, rs10244329, and rs11761556 and four SNPs in *LEPR* gene – rs12062820, rs1186403, rs1137101, and rs7516341. We based the selection on the known genetic linkage in both genes, according to HapMap Phase 3 genetic linkage information (*http://www.hapmap.org*). The SNPs were regarded as proxies for neighboring SNPs, when their pairwise r^2^ values exceeded 0.80. The set of most representative tagSNPs for *LEP* and *LEPR* gene regions was obtained using the Tagger algorithm available through the Haploview software (version 4.2) (14). To maximize statistical power, only the SNPs with minor allele frequency values exceeding 0.05 were analyzed.

Genomic DNA was isolated from the peripheral blood samples using QIAamp DNA Mini Kit (Qiagen, Hilden, Germany). SNPs genotyping was carried out by real time polymerase chain reaction (PCR) method on 7000 Sequence Detection System (Applied Biosystems, Foster City, CA, USA) using KASPar SNP genotyping chemistry (KBioscience Ltd, Hoddesdon, United Kingdom), which is based on allele-specific PCR amplification. The reactions were performed in accordance with manufacturer’s protocol. The allelic discrimination analysis was performed using SDS Software Version 1.2 (Applied Biosystems). Genotype assignment was performed and interpreted independently by two investigators.

### Statistical analysis

All calculations and statistical analyses were carried out in R statistical environment (R version 2.15.0, available from: *http://cran.r-project.org/*). Hardy-Weinberg test was used for normality testing. Where appropriate, the *P* values were adjusted for multiple testing, according to Bonferroni. Only the values exceeding the threshold of 0.05 after correction for multiple testing were considered significant. Relationships between genotype information and disease affection status were initially investigated separately for each SNP. Here, significance of association was estimated using χ^2^ test, and odds ratio values (OR) with 95% confidence intervals (CI) were calculated using functions in the gap package for R (*http://cran.r-project.org/web/packages/gap/index.html*). Associations were considered significant when the *P* value was <0.05.

Our genotype distribution was compared with those predicted by the Hardy-Weinberg equilibrium using χ^2^ goodness-of-fit test, as additional level of genotyping quality control. As the study participants were not related, haplotype reconstruction was done *in silico* using software tools for haplotype prediction. When referring to haplotypes, we considered the definition of haplotypes as a set of variants present in block on the same chromosome. We used SNPs to define specific haplotypes for both *LEP* and *LEPR* gene. Each haplotype is labeled as the sequence of alleles captured in the selected haplotype. As an example – we shortened the naming for haplotype in *LEPR* gene with rs1137101-G/rs7516341-T/rs1186403-C/rs12062820-C allele combination to GTCC. Haplotype frequencies and their associations with disease IRSA status were analyzed using haplo.stats statistical package (*http://mayoresearch.mayo.edu/mayo/research/schaid_lab/software.cfm*), which calculates indirectly measured haplotypes, under the assumption that study participants are not related and linkage phase is unknown. Only haplotypes with simulated frequencies below 0.05 were used in downstream analyses. Corrections for multiple testing were performed according to Benjamini Hochberg false discovery rate approach.

We estimated the power of our study to detect variations with low effect size. The power of detecting a significant result in the presence of actual genotype-phenotype effect with genotype relative risk equal to at least 2.0, was 86%, when taking into account the sample size included, significance threshold of 0.05, prevalence of IRSA in general population equal to 0.01, disease allele frequency of at least 10%, and considering multiplicative model of genetic association.

## Results

### Genotyping results

Genotype frequencies of investigated polymorphisms in both the study and control group were in accordance with those predicted by the Hardy-Weinberg equilibrium (*P* > 0.05). Genotype and allelic distribution of the *LEP* and *LEPR* polymorphisms are shown in Table 2.

**Table 2:** Genotype and allelic distribution of leptin (*LEP*) and leptin receptor (*LEPR*) polymorphisms of patients with and without idiopathic recurrent spontaneous abortion (IRSA)

	Allelic frequencies				Genotype frequencies					
	minor allele	MAF* controls	MAF cases	*P*	dominant model			recessive model		
*LEP*					*P*	odds ratio	95% confidence interval	*P*	odds ratio	95% confidence interval
rs7799039	A	0.40	0.46	0.16	0.534	0.86	0.54-1.37	0.099	1.58	0.92-2.77
rs21226227	T	0.08	0.98	0.42	0.805	1.22	0.18-10.66	0.422	0.78	0.42-1.43
rs11761556	C	0.45	0.42	0.52	0.714	0.90	0.51-1.59	0.504	1.17	0.73-1.90
rs10244329	A	0.46	0.50	0.37	0.886	0.96	0.58-1.61	0.173	1.45	0.85-2.51
*LEPR*										
rs1137101	A	0.48	0.54	0.09	0.472	0.83	0.50-1.39	0.048	1.66	1.00-2.80
rs7516341	C	0.33	0.40	0.05	0.034	0.61	0.38-0.97	0.354	1.40	0.68-3.00
rs1186403	C	0.28	0.23	0.10	0.315	1.26	0.80-2.00	0.082	0.51	0.23-1.10
rs12062820	T	0.18	0.18	1.00	0.657	0.79	0.26-2.39	0.847	0.95	0.59-1.54

*Minor allele frequency.

We found a higher allele frequency for rs7516341 (*P* = 0.052) in *LEPR* gene in the IRSA group. Also, the IRSA group had more frequent genotype distribution of rs7516341 (*P* = 0.034, OR 0.61, 95% CI 0.38-0.97) and rs1137101 (*P* = 0.048, OR 1.66, 95% CI 1.00-2.80) in *LEPR* gene. After adjustment for multiple testing according to Bonferroni, differences in allele and genotype frequencies failed to reach adjusted significance threshold. We did not find any significant association between rs12062820 and rs1186403 polymorphisms in *LEPR,* as well as rs7799039, rs2122627, rs10244329, and rs11761556 polymorphisms in *LEP* gene and IRSA.

### Haplotype analyses at LEP and LEPR loci

We identified 4 inferred haplotypes in *LEP* gene and 6 inferred haplotypes in the *LEPR* gene – the haplotypes identified are denoted with corresponding allele of the interrogated SNP (Table 3). Simulated *P* values reflecting the difference in haplotype frequency distribution are also presented. Haplotypes with frequency below 5% in the set of all cases were excluded from downstream analyses.

**Table 3:** Frequencies and distribution of probable haplotypes in patients with and without idiopathic recurrent spontaneous abortion (IRSA) as predicted by haplo.stats

rs7799039	rs2122627	rs11761556	rs10244329	Frequency in patients	Frequency in controls	Simulated *P*
G	T	C	A	0.05019	0.07931	0.26533
G	C	C	T	0.33186	0.36897	0.32593
G	C	A	T	0.12048	0.14254	0.35734
A	C	A	A	0.40024	0.38284	0.68377

rs1137101	rs7516341	rs1186403	rs12062820	Frequency in patients	Frequency in controls	Simulated *P*
G	T	C	C	0.05682	0.12851	0.0087
G	T	T	C	0.21407	0.22973	0.3936
A	T	T	T	0.11096	0.13368	0.500
G	C	C	C	0.13213	0.11630	0.82219
A	T	T	C	0.18268	0.16895	0.3940
A	C	T	C	0.16275	0.09096	0.0146

In patients with IRSA, GTCC haplotype in the *LEPR* gene locus was significantly less frequent than in controls (*P* = 0.00865, OR 0.45), contrary to ACTC haplotype (*P* = 0.0087, OR 1.98). We did not find a significant difference in the frequencies of the 4 most frequent haplotypes for the 4 analyzed SNPs in the *LEP* gene between the study and control group.

## Discussion

We showed that genetic variability in the *LEPR* gene was associated with IRSA. *LEP* and *LEPR* expression in various maternal tissues, placenta, and fetal tissues suggests the physiologic and pathophysiologic significance of leptin and leptin receptors in normal pregnancy (15-17). The relationship between *LEPR* expression and a successful embryo implantation has been shown by Allegra et al (18). They showed that *LEPR* may have a role in embryo-maternal cross-talk during the implantation window; endometrial *LEPR* expression was lower in patients with implantation failure. Also, previous studies reported significantly lower leptin concentrations in women who miscarried than in those with successful pregnancy, suggesting that leptin may play a role in preventing miscarriage (19-22). Leptin is capable of influencing placental nutrient transport, crucial for fetal growth regulation (23). Placental villi fragments have functional leptin receptors, which stimulates the system A of placental aminoacid transport through Janus kinase and signal transducer and activator of transcription proteins signaling pathway. Placental amino acids transport is reduced in pregnancies with fetal growth retardation (24,25). Genetic variability in the *LEP* and *LEPR* gene was previously associated with susceptibility to obesity and obesity-related metabolic diseases (26,27). The link between maternal obesity and pregnancy complications, including the risk of recurrent miscarriage, has also been reported (3). Furthermore, women with the *LEPR* 223A/G or 223G/G genotypes had an increased risk of developing severe preeclampsia compared with women with the 223A/A genotype, whereas 2548 G/A *LEP* polymorphism was associated with gestational diabetes mellitus (12,13). These polymorphisms are connected either with inadequate signaling capacity of leptin receptor and susceptibility to development of leptin resistance or with low leptin expression (due to mutation in gene promoter) and consequently low leptin response. Chin et al (28) failed to demonstrate any significant association of those polymorphisms in the *LEPR* and *LEP* gene with recurrent pregnancy loss. The polymorphisms investigated in our study are either in intron (rs7516341) or coding region (rs1137101) of *LEPR* gene and both can be connected with inefficient response to circulating leptin levels.

One of the limitations of our study is that, considering the number of samples surveyed, we attained sufficient power to detect only associations with larger effects (with effect size greater than 2.0), while association of variants with minor effects might have been missed. Another potential limitation lies in the SNP selection, which was aimed to capture most of the contributing genetic variation. We may still have missed rare functional polymorphisms or polymorphisms out of linkage with those tested in our study, particularly those in *LEP* gene. Also, we lacked the cytogenetic data on miscarried fetuses and polymorphism of fetal *LEP* and *LEPR* genes. Mutations in down-stream genes of fetal leptin signaling pathway are another possible pathologic mechanism behind RSA, but this was not assessed in our study. We also did not address the possibility of transplacental transport of maternal leptin and interaction with fetal leptin receptor, which might be further complicated by polymorphism on either side of interaction.

In conclusion, this study showed that genetic variability in the *LEPR* gene was associated with IRSA. Additional genetic association studies on a larger number of IRSA patients, as well as evaluation of other *LEPR* and *LEP* genetic variations, are necessary to elucidate the role of leptin signaling pathway in IRSA.
